# Factors associated with self-care behavior in patients with kidney failure receiving dialysis: a hospital-based cross-sectional study in Bandung, Indonesia

**DOI:** 10.1186/s12882-026-05196-2

**Published:** 2026-07-13

**Authors:** Mella Riski, Irma Melyani Puspitasari, Cherry Rahayu, Sofa D. Alfian

**Affiliations:** 1 Department of Pharmacology and Clinical Pharmacy, Faculty of Pharmacy, Universitas Padjadjaran, Jatinangor, 45363 Indonesia; 2 Center of Excellence for Pharmaceutical Care Innovation, Universitas Padjadjaran, Jatinangor, 45363 Indonesia; 3 Dr. Hasan Sadikin Central General Hospital, Bandung, 40161 Indonesia; 4 Center for Health Technology Assessment, Universitas Padjadjaran, Jatinangor, 45363 Indonesia

**Keywords:** Chronic kidney disease, Kidney failure, Dialysis, Factor, Self-care behavior

## Abstract

**Background:**

While self-care behaviors (SCB) are recognized as a crucial component of managing the adverse effects associated with chronic kidney disease (CKD), understanding of the factors influencing SCB remains limited, particularly in resource-constrained settings like Indonesia. This study aims to measure SCB scores and identify factors associated with SCB in patients with kidney failure receiving dialysis.

**Methods:**

Data collection was carried out at a tertiary hospital in Bandung, Indonesia, from September to November 2024. This study used three questionnaires that assessed SCB, health literacy (HL), and disease knowledge (DK) with a total of 43 items. The dependent variable was the SCB score. The independent variables included HL, DK, age, gender, marital status, education, income, occupation, comorbidities, body mass index (BMI), blood pressure, dialysis status, duration of dialysis, type of medication, route of drug administration, and number of medications. Data were analyzed using descriptive, univariate, and multivariate linear regression.

**Results:**

A total of 110 patients were enrolled, of whom 109 were receiving dialysis and one was not. The SCB score of patients with kidney failure receiving dialysis varied, with an average score of 57.5 ± 8.94 (range: 16–80). Factors significantly associated with high SCB scores were high DK (β = 7.992; *p* = 0.003), female (β = 4.235; *p* = 0.012), and income ≥ Indonesian RMW (Regional Minimum Wage) (β = 4.946; *p* = 0.029).

**Conclusions:**

A higher SCB score indicates an improvement in the patient’s SCB. DK, gender, and income were independently associated with SCB. Healthcare providers need to implement education and counseling to improve SCB by considering these characteristics.

**Supplementary Information:**

The online version contains supplementary material available at https://doi.org/10.1186/s12882-026-05196-2.

## Background

Chronic kidney disease (CKD) is a well-documented global health problem associated with increased cardiovascular risk, high morbidity and mortality rates, decreased quality of life, and escalating medical costs [1–3]. The staggering global burden of CKD, estimated at 697.5 million individuals in 2017, resulting in 1.2 million deaths [4], demands effective management strategies. Recent data from the Indonesian Health Survey (2023) indicates a prevalence of 0.18% in individuals aged ≥ 15 years and 0.2% in West Java [5]. Data from the Indonesian Renal Registry (IRR) (2020) attributes hypertension (35%) and diabetes (29%) as the primary causes of stage 5 CKD in Indonesia [6], suggesting a need for more targeted preventive measures. Furthermore, approximately 60% of ESRD patients in Indonesia receive dialysis [7].

Self-care behaviors (SCB) are commonly promoted as a means to alleviate the adverse effects of CKD [1]. However, the reliance on SCB as a primary management strategy warrants careful consideration. While adherence to therapy, dietary control, exercise, smoking cessation, and blood pressure monitoring are often cited as key SCB components [3], the effectiveness of these behaviors is contingent on complex factors, including patient knowledge, access to resources, and health literacy (HL) [8]. Existing research demonstrates a correlation between SCB and improved physical function, reduced urinary protein, and controlled blood pressure [9] but often fails to adequately address the socioeconomic and other barriers that may hinder SCB adaptation in diverse populations. Self-care in dialysis patients includes activities that support survival and maintain function, and it is essential for managing the condition, preventing complications, and improving quality of life [10].

Although limited HL is recognized as a predictor of poor SCB and adverse health outcomes [3, 11], the precise association between HL and SCB in CKD remains unclear. While Suarilah & Lin (2021) in Indonesia found that adequate HL indicates good SCB, the generalizability of these findings is limited by the study’s focus on early-stage CKD and the omission of disease knowledge (DK) as a contributing factor [12]. Furthermore, conflicting evidence from studies like Wong et al. (2018) [1], which found an inconsistent relationship between HL and SCB, highlights the need for a more nuanced understanding of the complex interplay of factors influencing SCB in CKD patients [1, 3].

The role of DK in promoting effective SCB is also inconclusive. While adequate DK is often cited as a prerequisite for informed decision-making and active engagement in disease management [3, 13], research by Gheewala et al. (2018) indicates that many CKD patients possess limited understanding of basic kidney information, raising questions about the effectiveness of knowledge-based interventions [14]. Although Tsai et al. (2021), a Taiwanese study, found a positive correlation between SCB and DK, its failure to consider the concurrent association between HL and SCB limits the study’s overall interpretability [2].

Despite the recognition that patient demographics, comorbidities, and laboratory data are associated with SCB [2, 3, 14], research examining the specific determinants of SCB within the Indonesian context remains scarce. Therefore, this study aims to address this gap by investigating the SCB score and to identify factors associated with SCB in patients with kidney failure receiving dialysis at a hospital in Bandung, Indonesia.

## Methods

### Study design and participants

This study is an observational study using a cross-sectional approach and was conducted in accordance with the Strengthening the Reporting of Observational Studies in Epidemiology (STROBE) guidelines. The study population consisted of patients with kidney failure receiving dialysis treated at a tertiary referral hospital in Bandung, West Java, Indonesia. The hospital provides comprehensive specialist and subspecialist services and serves as the highest referral center in West Java. Inclusion criteria were outpatients with CKD based on eGFR values in medical records, regardless of dialysis status, and adult patients ≥ 18 years. Patients with kidney transplants and pregnant women were excluded to reduce bias due to consideration of specific drug use. The sampling technique used was total sampling.

### Study variables

Andersen’s Behavioral Model is often used in research that analyzes SCB, comprising three factors: predisposing, enabling, and need factors as independent variables [15]. Predisposing factors are those that are associated with individuals’ use of health services, including age, gender, and social factors (e.g., education, occupation, and marital status), and health beliefs (HL and DK). Enabling factors considered to serve as conditions for service utilization include an individual’s income. The need factors are the patient’s perceived need for healthcare services and an evaluation of the patient’s medical condition, such as comorbidities, BMI, blood pressure, dialysis status, duration of dialysis, type of medication, route of drug administration, and number of medications. The SCB score was the dependent variable.

### Data collection

Data collection took place at a tertiary hospital in Bandung from September to November 2024. Patient characteristics were collected from the records room, while SCB, HL, and DK were collected through three adaptation questionnaires [1, 14, 16].

## Measurements

### Translation

The questionnaire was translated into Indonesian at the West Java Language Center to ensure that the translated items had the same meaning as the original items.

### Validity and reliability

A pilot test was conducted to test the questionnaire among 30 patients with kidney failure receiving dialysis at the same hospital. The aim of this pilot study was to determine whether there were any confusing questions and to illustrate the distribution of responses for each questionnaire [17].

The validity tests used were content and construct validity. Content validity ensures the instrument covers all essential items, assessed through literature review and expert evaluation [18]. The use of three experts is consistent with established methodological guidance for content validity assessment. According to Davis LL (1992) and Polit et al. (2007), a minimum of three experts is acceptable, and when three experts are used, a Content Validity Ratio (CVR) threshold of ≥ 0.78 [19] is recommended for item-level agreement, while a CVI ≥ 0.80 indicates acceptable content validity [20]. Content validity assessment is not recommended with fewer than three experts, as reliability becomes unstable [19]. Therefore, no alternative cut-off thresholds were applied in this study. Items with a CVI below 0.70, even after revision, may be considered for removal [19].

Construct validity refers to how well the questionnaire transforms [18]. Construct validity is used to evaluate questionnaires designed to measure constructs that cannot be directly observed. Correlation is used to test expected patterns of relationships. Analysis was conducted using Pearson product-moment and point-biserial correlation. An item is considered valid if the calculated r value is greater than the tabulated r [17].

Reliability testing was conducted to determine the consistency between items in a questionnaire. Reliability evaluation can be performed using Cronbach’s alpha and KR-20. An acceptable criterion is > 0.70 as an adequate measure of the reliability or internal consistency of an instrument [21, 22].

### Patient characteristics

Several patient characteristics were obtained: age (18–29; 30–49; or ≥ 50 years), gender (female/male), marital status (single, married, divorced, or widowed), education (elementary school, junior high school, high school, or university), income (regional minimum wage (RMW) Bandung, West Java, Indonesia: US$ 246.32) (< RMW or ≥ RMW), working status (yes/no), comorbidities (diabetes, hypertension, or both), body mass index (BMI) (< 18.5; 18.5–24.9; 25.0–29.9 or ≥ 30 kg/m^2^), blood pressure (< 120/<80; 120–139/80–89; 140–159/90–99; ≥ 160/ ≥ 100 mmHg), dialysis status (non-dialysis, HD, or CAPD), duration of dialysis (< 6; 6–12; or > 12 months), the type of medication in the last 3 months (antihypertensive; antihypertensive and other renal therapy; or antihypertensive, antidiabetic, and other renal therapy), the route of drug administration (peroral or peroral and insulin sc), and the number of medications in the last 3 months (< 3, 3–5, or > 5).

### Health literacy

The HL questionnaire developed by Chew et al. (2008) has been used in one study, which found that HL may not be directly associated with SCB and could be mediated by other factors, such as disease knowledge. Health literacy was measured using a health literacy questionnaire, consisting of three questions: How confident are you when filling out hospital forms?; How often do others help you read hospital documents? (For example, doctor’s information and drug information labels); How often do you have difficulty learning about medical conditions due to difficulty reading hospital documents? (For example, doctor’s information and drug information labels) [1]. Each statement is on a scale of 0–4 [1]. Scores for each item are summed and divided by 3. Limited health literacy is considered if the score is ≥ 6 out of a maximum score of 12 [1].

### Disease knowledge

The disease knowledge questionnaire developed by Gheewala et al. (2018) has not yet been applied in other studies, although it is capable of providing an overview of patients’ disease-related knowledge. The CKD knowledge questionnaire consists of 24 questions with correct (score 1), incorrect (score 0), or don’t know answers (score 0) [14]. Example question: “A person can live a normal life with one healthy kidney.” A patient’s disease knowledge is considered adequate if at least 50% of the answers are correct [14].

### Self-care behavior

The CKDSC instrument developed by Wang et al. (2019) has been utilized in two subsequent studies [3, 23] examining factors associated with self-care behavior (SCB) among patients with CKD, where factors such as age, occupation, smoking status, dialysis status, comorbidities, and health literacy (HL) were identified, and higher HL was associated with better SCB. The self-care behavior questionnaire consists of five domains: adherence (5 items), diet (4 items), exercise (3 items), smoking habits (2 items), and blood pressure monitoring (2 items) [16]. Scores are based on a Likert scale ranging from 1 (never) to 5 (always) [16]. The adherence domain uses negative statements; therefore, scores are reverse-coded. The total score is obtained by summing each item, ranging from 16 to 80 points. Higher scores indicate better SCB [16].

### Data analysis

Descriptive analysis was used to summarize the distribution of variables as frequency (N) and percentage (%). Univariate linear regression was performed to assess the effect of each independent variable on SCB, and variables with *p* < 0.25 were included in the multivariate model [24]. Prior to multivariate analysis, classical assumption tests were conducted, including normality, heteroscedasticity, and multicollinearity [25–27]. Multivariate linear regression was then applied to examine the association between independent variables and SCB, with *p* < 0.05 considered statistically significant [24]. Statistical analysis was performed using IBM SPSS Statistics, Version 27.0 (IBM Corp., Armonk, NY, USA).

### Ethical considerations

This study has obtained ethical clearance from the Universitas Padjadjaran Ethics Committee. Number: 719/UN6.KEP/EC/2024. Information related to the study was provided to patients in accordance with ethical requirements.

## Results

### Validity and reliability

The content validity of the questionnaire was conducted twice. The CVI’ results for the content validity of the first questionnaire, namely the health literacy questionnaire, were 0.557; the knowledge questionnaire was 0.944; and the self-care behavior questionnaire was 0.747. The content validity of the second questionnaire was obtained with a CVI’ value of 1 for all questionnaires. These results indicate that the three questionnaires are valid because the CVI’ value is > 0.80 [20].

The results of the construct validity test obtained showed that all items in the three questionnaires had a calculated r value greater than the r Table (0.361). Based on the reliability analysis, Cronbach’s alpha values for the health literacy questionnaire and the self-care behavior questionnaire were 0.711 and 0.818, respectively, while the KR-20 value for the knowledge questionnaire was 0.822. The results of the analysis with both Cronbach’s alpha and KR-20 showed that all questionnaires were reliable because they had values > 0.7.

### Patient characteristics

**Fig. 1:**
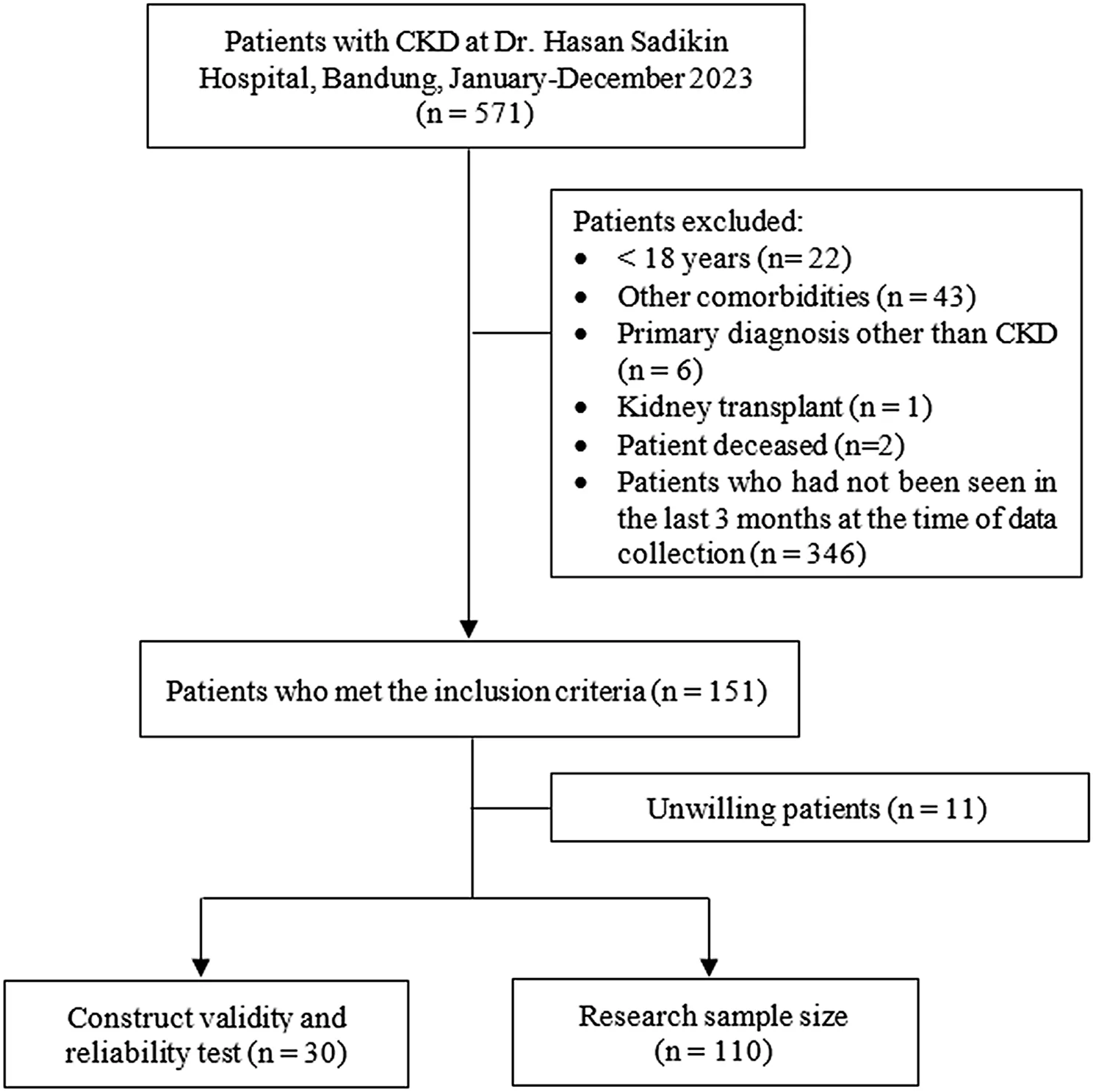
Flowchart of the study sample

This study screened 571 patients with CKD, with 151 meeting inclusion criteria. After 11 patients declined, 140 were included. Thirty patients were included for construct validity and reliability tests, resulting in a final sample size of 110 patients who were enrolled, of whom 109 were receiving dialysis and one was not (Fig. 1).

The characteristics of patients with kidney failure receiving dialysis are shown in Table 1. The results show that the majority of patients were aged 30–49 years (52.7%), female (50.9%), married (78.2%), had a high school education (42.7%), had an income less than minimum wage (79.1%), were unemployed (57.3%), had comorbid hypertension (75.5%), BMI of 18.5–24.9 kg/m² (52.7%), had blood pressure ranging from 140–159/90–99 mmHg (33.6%), underwent hemodialysis (80%), had a dialysis duration of more than 12 months (95.4%), used antihypertensive medications and other renal therapies (84.5%), and administered oral medications (98.2%), and received 3–5 medications (63.6%). 

**Table 1:** Distribution of characteristics, HL, and DK among patients

Characteristics	*N*	%
Age 18–29 years 30–49 years ≥ 50 years	13 58 39	11.8 52.7 35.5
Gender Male Female	54 56	49.1 50.9
Marital Status Single Married Divorced Widowed	15 86 7 2	13.6 78.2 6.4 1.8
Education Elementary School Junior High School High School University	16 18 47 29	14.5 16.4 42.7 26.4
Income < RMW (< Rp. 4.048.463) ≥ RMW (≥ Rp. 4.048.463)	87 23	79.1 20.9
Occupation Unemployed Employed	63 47	57.3 42.7
Comorbidities None Hypertension Diabetes Hypertension and Diabetes	16 83 5 6	14.5 75.5 4.5 5.5
BMI < 18,5 kg/m^2^ 18,5–24,9 kg/m^2^ 25–29,9 kg/m^2^ ≥ 30 kg/m^2^	18 58 29 5	16.4 52.7 26.4 4.5
Blood Pressure < 120/<80 mmHg 120–139/ 80–89 mmHg 140–159/ 90–99 mmHg ≥ 160/ ≥ 100 mmHg	12 31 37 30	10.9 28.9 33.6 27.3
Dialysis Status Non-dialysis Hemodialysis CAPD	1 88 21	0.9 80.0 19.1
Duration of Dialysis < 6 months 6– 12 months > 12 months	1 4 104	0.9 3.7 95.4
Type of Medication Antihypertensive Other Renal Therapy Antihypertensive and Other Renal Therapy Antihypertensive, Antidiabetic, and Other Renal Therapy	6 9 93 2	5.5 8.2 84.5 1.8
Route of Drug Administration Oral Oral and subcutaneous insulin	108 2	98.2 1.8
The number of Medications < 3 3–5 > 5	10 70 30	9.1 63.6 27.3
Health literacy Limited (≥ 6) Adequate (< 6)	26 84	23.6 76.4
Disease knowledge Low (< 50%) High (≥ 50%)	11 99	10 90

RMW: Regional Minimum Wage

CAPD: Continuous Ambulatory Peritoneal Dialysis

### Health literacy

Table 1 shows that 76.4% of patients had adequate health literacy and 23.6% had limited health literacy. This indicates that the majority of patients had adequate health literacy.

### Disease knowledge

The results (Table 1) show that 90% of patients had high disease knowledge and 10% had low disease knowledge. This indicates that most patients with kidney failure receiving dialysis have high levels of knowledge.

### Self-care behavior

Table 2 shows that the average SCB score for 110 patients was 57.5 ± 8.94. This result is close to the results of previous research, which showed an average SCB score of 62.12 ± 8.76 [16]. The SCB score ranges from 16 to 80. The higher the SCB score, the higher the patient’s self-care behavior.

**Table 2:** Distribution of patient responses to each item of the SCB questionnaire

Item	Average ± SD	Median (Min; Max)
**Medication Adherence**	**22.02 ± 2.62**	**23 (14; 25)**
I changed the prescribed dosage times.	4.2 ± 0.97	5 (1; 5)
I stopped taking my medication.	4.53 ± 0.86	5 (2; 5)
I did not take my medication regularly.	4.27 ± 1.03	5 (1; 5)
I changed the frequency of my prescribed medication doses.	4.05 ± 0.99	4 (2; 5)
I changed the prescribed dosage.	4.97 ± 0.16	5 (4; 5)
**Diet Control**	**14.55 ± 3.64**	**14 (7; 20)**
I adhere to the principles of a kidney disease diet when eating.	3.64 ± 1.15	4 (1; 5)
I consume adequate amounts of food based on the recommendations of a healthcare professional.	3.63 ± 1.23	3,5 (1; 5)
I control my food and nutrition intake.	3.7 ± 1.19	4 (1; 5)
I adhere to the principles of a kidney disease diet even when on vacation or eating out.	3.58 ± 1.44	4 (1; 5)
**Exercise**	**7.08 ± 2.91**	**7 (3; 15)**
I exercise regularly.	2.9 ± 1.27	3 (1; 5)
I try to exercise to manage my kidney disease even when I do not feel like it.	2.11 ± 1.14	2 (1; 5)
I make time to exercise even when I’m busy.	2.07 ± 1.23	2 (1; 5)
**Smoking Habits**	**8.39 ± 2.17**	**9 (2; 10)**
I do not smoke every day.	4.72 ± 0.84	5 (1; 5)
I avoid people or areas where smoking occurs.	3.66 ± 1.6	4 (1; 5)
**Blood Pressure Monitoring**	**5.47 ± 3.22**	**6 (2; 10)**
I monitor my blood pressure.	2.62 ± 1.63	2 (1; 5)
I can increase the frequency of my blood pressure monitoring, especially when I’m feeling unwell.	2.85 ± 1.74	3 (1; 5)
**Total SCB Scores**	**57.5 ± 8.94**	**57 (42; 75)**

### Analysis of factors associated with SCB

The results of the univariate linear regression test (Table 3) showed that the independent variables with a p-value < 0.25 on total SCB, the domain of adherence, diet, exercise, smoking habits, and blood pressure monitoring.

**Table 3:** Univariate linear regression analysis of HL, DK, and patient characteristics on SCB

Independent Variable	Total SCB	Adherence	Diet	Exercise	Smoking Habits	Blood Pressure Monitoring
	*p*-value					
HL	0.193^*^	0.053^*^	0.845	0.313	0.039^*^	0.643
DK	< 0.001^*^	0.065^*^	0.066^*^	0.082^*^	0.002^*^	< 0.001^*^
Age	0.793	0.185^*^	0.147^*^	0.052^*^	0.276	0.332
Gender	0.040^*^	0.277	0.183^*^	0.412	< 0.001^*^	0.132^*^
Marital Status	0.338	0.238^*^	0.448	0.465	0.771	0.088^*^
Education	< 0.001^*^	0.037^*^	0.762	0.167^*^	0.036^*^	< 0.001^*^
Income	0.002^*^	0.116^*^	0.545	0.003^*^	0.438	< 0.001^*^
Occupation	0.615	0.216^*^	0.254	0.016^*^	0.004^*^	0.522
Comorbidities	0.697	0.365	0.529	0.035^*^	0.871	0.598
BMI	0.972	0.491	0.278	0.127^*^	0.117^*^	0.172^*^
Blood Pressure	0.596	0.836	0.286	0.787	0.704	0.012^*^
Dialysis Status	< 0.001^*^	0.556	0.606	0.103^*^	0.062^*^	< 0.001^*^
Duration of Dialysis	0.630	0.225^*^	0.412	0.950	0.697	0.804
Type of Medication	0.153^*^	0.362	0.464	0.398	0.269	0.328
Routes of Drug Administration	0.382	0.992	0.684	0.349	0.465	0.119^*^
The number of Medications	0.912	0.882	0.367	0.353	0.512	0.662

*p-value < 0.25

The classical assumption tests showed normal distribution (*p* > 0.05) and no multicollinearity (tolerance > 0.100; VIF < 10.00) in the total SCB and the adherence, dietary, physical activity, smoking behavior, and blood pressure monitoring domains. Heteroscedasticity was identified in the total SCB, adherence, and smoking behavior domains (*p* < 0.05); therefore, HC3 adjustment was applied [28]. Based on multivariate linear regression analysis (Table 4), DK (β = 7.992; *p* = 0.003), female (β = 4.235; *p* = 0.012), and income ≥ RMW (regional minimum wage) (β = 4.946; *p* = 0.029) are significantly associated with high SCB scores.

**Table 4:** Multivariate linear regression analysis of characteristics, DK, HL on SCB

Variable	Total SCB^a^		Adherence^a^		Diet		Exercise		Smoking Habits^a^		Blood Pressure Monitoring	
	β	*p*-value	β	*p*-value	β	*p*-value	β	*p*-value	β	*p*-value	β	*p*-value
HL Limited (Ref.) Adequate	0.679	0.678	1.318	0.036*	-	-	-	-	0.774	0.143	-	-
DK Low (Ref.) High	7.992	0.003*	0.413	0.568	1.879	0.105	1.711	0.083	1.641	0.034*	1.688	0.040*
Age 18–29 years (Ref.) 30–49 years ≥ 50 years	-	-	-0.349 0.507	0.724 0.616	-0.034 0.973	0.976 0.402	-0.548 -1.130	0.551 0.272	-	-	-	-
Gender Male (Ref.) Female	4.235	0.012*	-	-	0.844	0.221	-	-	0.993	0.030*	1.143	0.021*
Marital Status Single (Ref.) Married Divorced Widowed	-	-	0.139 -3.468 0.086	0.885 0.025* 0.981	-	-	-	-	-	-	-0.395 -2.701 -0.245	0.632 0.025* 0.902
Education Elementary School (Ref.) Junior High School High School University	0.423 3.603 3.653	0.861 0.168 0.206	0.052 0.704 1.405	0.955 0.343 0.127	-	-	-1.044 -0.454 -0.818	0.315 0.619 0.465	0.552 0.450 0.909	0.461 0.542 0.281	0.874 3.257 3.482	0.313 < 0.001* < 0.001*
Income < RMW (Ref.) ≥ RMW	4.946	0.029*	1.118	0.184	-	-	1.351	0.130	-	-	1.059	0.137
Occupation Unemployed (Ref.) Employed	-	-	-1.088	0.060	-	-	0.624	0.364	-0.950	0.073	-	-
Comorbidities None (Ref.) Hypertension Diabetes Hypertension and diabetes	-	-	-	-	-	-	-0.282 -0.338 -1.977	0.734 0.828 0.185	-	-	-	-
BMI < 18,5 kg/m^2^ 18,5–24,9 kg/m^2^ (Ref.) 25,0–29,9 kg/m^2^ ≥ 30 kg/m^2^	-	-	-	-	-	-	0.239 0.482 2.225	0.767 0.481 0.152	0.560 -0.689 0.965	0.205 0.200 0.433	-0.849 0.052 0.783	0.226 0.930 0.555
Blood Pressure <120/<80 mmHg (Ref.) 120–139/80–89 mmHg 140–159/90–99 mmHg ≥ 160/ ≥ 100 mmHg	-	-	-	-	-	-	-	-	-	-	0.052 -1.216 0.378	0.954 0.154 0.688
Dialysis Status Non-dialysis (Ref.) Hemodialysis CAPD	1.881 0.526	0.734 0.359	-	-	-	-	1.874 1.863	0.569 0.583	-0.683 0.218	0.886 0.964	2.515 4.262	0.362 0.140
Duration of Dialysis < 6 months (Ref.) 6–12 months > 12 months	-	-	-4.475 -3.129	0.022* 0.003*	-	-	-	-	-	-	-	-
Type of Medication Antihypertensive (Ref.) Other Renal Therapy Antihypertensive, and Other Renal Therapy Antihypertensive, Antidiabetic, and Other Renal Therapy	-4.789 -0.905 -1.651	0.271 0.750 0.696	-	-	-	-	-	-	-	-	-	-
Route of Drug Administration Oral Oral and subcutaneous insulin	-	-	-	-	-	-	-	-	-	-	1.688	0.040*

*p-value < 0.05

^a^Multivariate linear regression analysis with HC3

RMW: Regional Minimum Wage

CAPD: Continuous Ambulatory Peritoneal Dialysis

## Discussion

The findings of this study revealed that the mean SCB score was 57.5, suggesting that this result is comparable to prior study findings of 62.12 [16]. An elevated SCB score indicates an improvement in the patient’s self-care behavior (SCB). DK demonstrated a significant positive correlation with high SCB scores, suggesting that knowledge about the disease may play a role in shaping patients’ engagement in SCB. Furthermore, gender and income were also significantly associated with high SCB scores, indicating that sociodemographic characteristics contribute to variations in patients’ ability to perform effective SCB. In contrast, despite most patients having adequate HL, HL did not show a significant correlation with SCB. Moreover, education, dialysis status, and type of medication demonstrate no significant association with SCB.

The results of this study indicate that the mean SCB score in patients with kidney failure receiving dialysis is almost identical to previous studies. Yu et al.‘s (2021) study obtained an average SCB score of 61.0, while Wang et al.‘s (2019) study obtained an average SCB score of 62.12. SCB refers to the ability to learn and practice behaviors that enable patients to better manage CKD [1]. Other studies using different instruments, including Avanji et al. (2021) and Noviana and Zahra (2012), demonstrated good self-care behavior (SCB) among patients with kidney failure receiving dialysis.

We observed patients with high DK scored 7.992 points higher on the SCB score than those with low DK. Similar results were found in a study by Tsai et al. (2021), which demonstrated that higher levels of knowledge were associated with better SCB [29]. Adequate DK reflects the ability to apply problem-solving strategies and seek information by reading health information or discussing with healthcare professionals [30]. Low DK may be associated with the number of patient visits to healthcare providers and a lack of education regarding CKD [29]. Research examining the relationship between DK and SCB among patients with kidney failure receiving dialysis remains limited. Furthermore, we observed that female patients scored significantly higher on the SCB score, which is similar to the study by Wang et al. (2019). This may be because females tend to be more sensitive to responses from family and relatives, thus influencing self-management compared to men [31].

Our findings showed that patients with incomes ≥ RMW scored higher than those with incomes < RMW. This finding is similar to research by Suarilah & Lin (2022), which showed that income is one factor influencing SCB [12]. A monthly income at or above the regional minimum wage was significantly associated with overall self-management and problem-solving skills [12]. Another study by Van Duong et al. (2019) found that patients with insufficient monthly income experienced difficulties in addressing daily challenges, both in terms of understanding and systematic approaches [32].

We further found that HL was not significantly associated with SCB score. This result is similar to studies by Wong et al. (2018) and Schrauben et al. (2020), which found no correlation between HL and SCB in CKD patients. However, other studies have shown a positive correlation between HL and SCB [3, 12, 23, 33]. The inconsistency of some studies may be due to their focus solely on the correlation between functional HL (such as reading and writing) and SCB [34]. Of note, health-related reading and writing skills are not always associated with understanding in decision-making [35]. The association between HL and SCB in patients with kidney failure receiving dialysis has not been extensively studied.

However, we observed that education was not significantly associated with SCB score. This finding is consistent with Kim and Cho (2021), who reported that education was not significantly associated with the SCB score in patients undergoing dialysis. However, other studies have shown a significant association between education and SCB [3, 12, 16, 30, 36]. The inconsistency in research results may be due to differences in other patient characteristics, such as age [37] or the association of DK [29].

Hemodialysis and CAPD patients scored higher than non-dialysis patients. However, these results were not significantly associated. The low SCB scores in early-stage CKD patients or those not on dialysis may be due to the fact that these patients do not yet exhibit physical symptoms of kidney damage and therefore lack an understanding of CKD [23]. ESRD patients experience deficient erythropoietin production and decreased red blood cell production, resulting in anemia and physical symptoms due to gradual uremia [16]. Therefore, ESRD patients on dialysis tend to have a higher awareness of the importance of routinely managing their CKD and better SCB compared to early-stage CKD patients not on dialysis [23].

Patients receiving other renal therapy; antihypertensive drugs and other renal therapy; and antihypertensive drugs, antidiabetics, and other renal therapy had lower scores compared to patients receiving antihypertensive drugs. The results showed that medication type was not significantly associated with SCB score. Other studies have shown an association between medication type and therapy adherence but did not analyze the correlation with the SCB score [38]. The type of medication used is associated with side effects [38] and the frequency of medication use [39], which can be associated with therapy adherence. However, adherence may be associated with other factors, such as dialysis duration. Our study demonstrated that dialysis duration was significantly associated with adherence domains, and the results are similar to research by Tayebi et al. (2019) [40].

This study found that several factors were associated with SCB in patients with kidney failure receiving dialysis. The factors significantly associated with SCB included DK, gender, and income. Patient involvement in SCB is one way to mitigate the negative impacts associated with CKD. Healthcare providers can provide regular education regarding CKD management to patients, taking into account patient characteristics, particularly those with low DK, male gender, and income < RMW. Previous research has shown that a 6-month educational intervention demonstrated better clinical outcomes [41]. Educational interventions include therapy adherence, dietary control, exercise, smoking habits, and blood monitoring [3]. Furthermore, education regarding CKD knowledge, especially in patients with low DK, is expected to improve SCB. Healthcare providers can also consider counseling patients to understand their understanding and challenges related to CKD management. Regular communication between patients and healthcare providers is expected to improve SCB [3].

This study possesses several strengths. Firstly, it directly addresses the factors associated with SCB in patients with kidney failure receiving dialysis, an area that requires greater attention in Indonesia. The findings can inform healthcare providers, enabling them to tailor interventions and support systems to better address the specific needs and characteristics of their patients, potentially enhancing their capacity for effective disease management. Secondly, the use of total sampling, while pragmatic, offers a comprehensive snapshot of the factors influencing SCB within the specific population studied.

However, several limitations must be acknowledged. First, the limited representation of non-dialysis CKD patients and the study’s reliance on data from a single hospital restrict the generalizability of the findings. Second, the cross-sectional study design precludes the establishment of causal relationships between the identified factors and SCB. Furthermore, the absence of nephrologists and dialysis nurses in the content validity panel may have limited the breadth of clinical perspectives. Finally, the reliance on self-reported questionnaires introduces the potential for recall bias and social desirability bias, which could influence the accuracy and validity of the data. Participants may have difficulty accurately recalling past behaviors, or they may provide responses that they perceive as more socially acceptable rather than reflecting their true behaviors. The confluence of these limitations warrants caution in interpreting the results and highlights the need for further research employing more robust methodologies and broader sampling frames.

Future research should investigate the association of social support networks and patients’ perceptions of kidney failure severity and treatment efficacy with SCB. Furthermore, rigorously designed intervention studies are needed to evaluate the effectiveness of culturally tailored educational programs in enhancing SCB and improving clinical outcomes in Indonesian patients with kidney failure receiving dialysis.

## Conclusion

An elevated SCB score indicates an improvement in the patient’s self-care behavior (SCB). High levels of disease knowledge, female gender, and having an income equal to or greater than RMW were significantly associated with high SCB scores. Healthcare providers can leverage these findings to implement tailored education and counseling programs for patients with kidney failure receiving dialysis, with particular attention to addressing knowledge gaps, recognizing potential gender-specific barriers, and providing resources to patients with lower incomes, thereby fostering improved SCB.

## Supplementary Information

Below is the link to the electronic supplementary material.

Supplementary Material 1

## Data Availability

The data presented in this study are available in the manuscript and supplementary materials.
