# Supplementary material for: Factors associated with self-care behavior in patients with kidney failure receiving dialysis: a hospital-based cross-sectional study in Bandung, Indonesia
Source: BMC Nephrol. 2026 Jul 13;27:594. doi: 10.1186/s12882-026-05196-2 (PMC13647733; doi:10.1186/s12882-026-05196-2)
Supplement: Supplementary file 1 [file 12882_2026_5196_MOESM1_ESM.docx]

**S1. The content validity test of the health literacy questionnaire**

| **Variable** | **Number of Item** | **Essential Item Assessment** | | | **CVR** | **Comment** | **Essential Item Assessment** | | | **CVR** | **Comment** |
| --- | --- | --- | --- | --- | --- | --- | --- | --- | --- | --- | --- |
|  |  | **Expert 1** | **Expert 2** | **Expert 3** |  |  | **Expert 1** | **Expert 2** | **Expert 3** |  |  |
| Health Literacy | 1 | - | - | $\surd$ | -0.33 | - What do you mean by "sure"? - This question is a bit confusing regarding the document in question and why you need to be sure when filling it out. - Add the statement "For example: information from a doctor, drug information label." | $\surd$ | $\surd$ | $\surd$ | 1 | - |
|  | 2 | $\surd$ | $\surd$ | $\surd$ | 1 | - | $\surd$ | $\surd$ | $\surd$ | 1 | - |
|  | 3 | $\surd$ | $\surd$ | $\surd$ | 1 | - | $\surd$ | $\surd$ | $\surd$ | 1 | - |
| Average CVR (CVI’) | **CVI’ = 0.557** | | | | | | **CVI’ = 1** | | | | |

CVR: Content Validity Ratio

CVI’: Content Validity Index

**S2. The content validity test of the disease knowledge questionnaire**

| **Variable** | | **Number of Item** | **Essential Item Assessment** | | | **CVR** | **Comment** | **Essential Item Assessment** | | | **CVR** | **Comment** |
| --- | --- | --- | --- | --- | --- | --- | --- | --- | --- | --- | --- | --- |
|  |  |  | **Expert 1** | **Expert 2** | **Expert 3** |  |  | **Expert 1** | **Expert 2** | **Expert 3** |  |  |
| Chronic Kidney Disease Knowledge | Section 1 | 1 | $\surd$ | $\surd$ | $\surd$ | 1 | - | $\surd$ | $\surd$ | $\surd$ | 1 | - |
|  |  | 2 | $\surd$ | $\surd$ | $\surd$ | 1 | - | $\surd$ | $\surd$ | $\surd$ | 1 | - |
|  |  | 3 | $\surd$ | $\surd$ | $\surd$ | 1 | - | $\surd$ | $\surd$ | $\surd$ | 1 | - |
|  | Section 2 | 4 | $\surd$ | $\surd$ | $\surd$ | 1 | - | $\surd$ | $\surd$ | $\surd$ | 1 | - |
|  |  | 5 | $\surd$ | $\surd$ | $\surd$ | 1 | - | $\surd$ | $\surd$ | $\surd$ | 1 | - |
|  |  | 6 | $\surd$ | $\surd$ | $\surd$ | 1 | - | $\surd$ | $\surd$ | $\surd$ | 1 | - |
|  |  | 7 | $\surd$ | $\surd$ | $\surd$ | 1 | - | $\surd$ | $\surd$ | $\surd$ | 1 | - |
|  |  | 8 | - | $\surd$ | $\surd$ | 0.33 | - | $\surd$ | $\surd$ | $\surd$ | 1 | - |
|  |  | 9 | - | $\surd$ | $\surd$ | 0.33 | - | $\surd$ | $\surd$ | $\surd$ | 1 | - |
|  | Section 3 | 10 | $\surd$ | $\surd$ | $\surd$ | 1 | - | $\surd$ | $\surd$ | $\surd$ | 1 | - |
|  |  | 11 | $\surd$ | $\surd$ | $\surd$ | 1 | - | $\surd$ | $\surd$ | $\surd$ | 1 | - |
|  |  | 12 | $\surd$ | $\surd$ | $\surd$ | 1 | - | $\surd$ | $\surd$ | $\surd$ | 1 | - |
|  |  | 13 | $\surd$ | $\surd$ | $\surd$ | 1 | - | $\surd$ | $\surd$ | $\surd$ | 1 | - |
|  | Section 4 | 14 | $\surd$ | $\surd$ | $\surd$ | 1 | - | $\surd$ | $\surd$ | $\surd$ | 1 | - |
|  |  | 15 | $\surd$ | $\surd$ | $\surd$ | 1 | - | $\surd$ | $\surd$ | $\surd$ | 1 | - |
|  |  | 16 | $\surd$ | $\surd$ | $\surd$ | 1 | - | $\surd$ | $\surd$ | $\surd$ | 1 | - |
|  |  | 17 | $\surd$ | $\surd$ | $\surd$ | 1 | - | $\surd$ | $\surd$ | $\surd$ | 1 | - |
|  |  | 18 | $\surd$ | $\surd$ | $\surd$ | 1 | - | $\surd$ | $\surd$ | $\surd$ | 1 | - |
|  |  | 19 | $\surd$ | $\surd$ | $\surd$ | 1 | - | $\surd$ | $\surd$ | $\surd$ | 1 | - |
|  | Section 5 | 20 | $\surd$ | $\surd$ | $\surd$ | 1 | - | $\surd$ | $\surd$ | $\surd$ | 1 | What does excess water mean? Laypeople worry they won't understand. |
|  |  | 21 | $\surd$ | $\surd$ | $\surd$ | 1 | - | $\surd$ | $\surd$ | $\surd$ | 1 | - |
|  |  | 22 | $\surd$ | $\surd$ | $\surd$ | 1 | - | $\surd$ | $\surd$ | $\surd$ | 1 | - |
|  |  | 23 | $\surd$ | $\surd$ | $\surd$ | 1 | - | $\surd$ | $\surd$ | $\surd$ | 1 | - |
|  |  | 24 | $\surd$ | $\surd$ | $\surd$ | 1 | - | $\surd$ | $\surd$ | $\surd$ | 1 | - |
| Average CVR (CVI’) | | **CVI’= 0.944** | | | | | | **CVI’ = 1** | | | | |

CVR: Content Validity Ratio

CVI’: Content Validity Index

**S3. The content validity test of the Self-Care Behavior Questionnaire**

| **Variable** | | **Number of Item** | **Essential Item Assessment** | | | **CVR** | **Comment** | **Essential Item Assessment** | | | **CVR** | **Comment** |
| --- | --- | --- | --- | --- | --- | --- | --- | --- | --- | --- | --- | --- |
|  |  |  | **Expert 1** | **Expert 2** | **Expert 3** |  |  | **Expert 1** | **Expert 2** | **Expert 3** |  |  |
| Chronic Kidney Disease Self-Care | Therapy Adherence | 1 | $\surd$ | $\surd$ | $\surd$ | 1 | - | $\surd$ | $\surd$ | $\surd$ | 1 | - |
|  |  | 2 | $\surd$ | $\surd$ | $\surd$ | 1 | - | $\surd$ | $\surd$ | $\surd$ | 1 | - |
|  |  | 3 | $\surd$ | $\surd$ | $\surd$ | 1 | - | $\surd$ | $\surd$ | $\surd$ | 1 | - |
|  |  | 4 | $\surd$ | $\surd$ | $\surd$ | 1 | - | $\surd$ | $\surd$ | $\surd$ | 1 | - |
|  |  | 5 | $\surd$ | $\surd$ | $\surd$ | 1 | - | $\surd$ | $\surd$ | $\surd$ | 1 | - |
|  | Diet Control | 6 | - | - | $\surd$ | - 0.33 | "What are the principles of diet?"  "Do you eat at home? Because item 9 also relates to eating." | $\surd$ | $\surd$ | $\surd$ | 1 | - |
|  |  | 7 | - | $\surd$ | $\surd$ | 0.33 | - | $\surd$ | $\surd$ | $\surd$ | 1 | - |
|  |  | 8 | - | $\surd$ | $\surd$ | 0.33 | - | $\surd$ | $\surd$ | $\surd$ | 1 | - |
|  |  | 9 | - | $\surd$ | $\surd$ | 0.33 | - | $\surd$ | $\surd$ | $\surd$ | 1 | - |
|  | Exercise | 10 | - | $\surd$ | $\surd$ | 0.33 | "What do you mean by regular?" | $\surd$ | $\surd$ | $\surd$ | 1 | - |
|  |  | 11 | $\surd$ | $\surd$ | $\surd$ | 1 | - | $\surd$ | $\surd$ | $\surd$ | 1 | - |
|  |  | 12 | $\surd$ | $\surd$ | $\surd$ | 1 | - | $\surd$ | $\surd$ | $\surd$ | 1 | - |
|  | Smoking Habits | 13 | $\surd$ | $\surd$ | $\surd$ | 1 | - | $\surd$ | $\surd$ | $\surd$ | 1 | - |
|  |  | 14 | $\surd$ | $\surd$ | $\surd$ | 1 | - | $\surd$ | $\surd$ | $\surd$ | 1 | - |
|  | Blood Pressure Monitoring | 15 | $\surd$ | $\surd$ | $\surd$ | 1 | - | $\surd$ | $\surd$ | $\surd$ | 1 | - |
|  |  | 16 | $\surd$ | $\surd$ | $\surd$ | 1 | - | $\surd$ | $\surd$ | $\surd$ | 1 | - |
| Average CVR (CVI’) | | **CVI’ = 0.747** | | | | | | **CVI’ = 1** | | | | |

CVR: Content Validity Ratio

CVI’: Content Validity Index

S4. The construct validity test of the health literacy questionnaire

| **Statement** | **r_value_** | **r_table_** | **Validity** |
| --- | --- | --- | --- |
| 1 | 0.607 | 0.361 | Valid |
| 2 | 0.924 | 0.361 | Valid |
| 3 | 0.826 | 0.361 | Valid |

S5. The construct validity test of the disease knowledge questionnaire

| **Question** | **r_value_** | **r_table_** | **Validity** |
| --- | --- | --- | --- |
| 1 | 0.442 | 0.361 | Valid |
| 2 | 0.579 | 0.361 | Valid |
| 3 | 0.489 | 0.361 | Valid |
| 4 | 0.413 | 0.361 | Valid |
| 5 | 0.473 | 0.361 | Valid |
| 6 | 0.423 | 0.361 | Valid |
| 7 | 0.504 | 0.361 | Valid |
| 8 | 0.444 | 0.361 | Valid |
| 9 | 0.465 | 0.361 | Valid |
| 10 | 0.417 | 0.361 | Valid |
| 11 | 0.390 | 0.361 | Valid |
| 12 | 0.370 | 0.361 | Valid |
| 13 | 0.364 | 0.361 | Valid |
| 14 | 0.390 | 0.361 | Valid |
| 15 | 0.431 | 0.361 | Valid |
| 16 | 0.504 | 0.361 | Valid |
| 17 | 0.375 | 0.361 | Valid |
| 18 | 0.423 | 0.361 | Valid |
| 19 | 0.406 | 0.361 | Valid |
| 20 | 0.432 | 0.361 | Valid |
| 21 | 0.391 | 0.361 | Valid |
| 22 | 0.382 | 0.361 | Valid |
| 23 | 0.661 | 0.361 | Valid |
| 24 | 0.432 | 0.361 | Valid |

S6. The construct validity test of the self-care behavior questionnaire

| **Statement** | **r_value_** | **r_table_** | **Validity** |
| --- | --- | --- | --- |
| 1 | 0.373 | 0.361 | Valid |
| 2 | 0.421 | 0.361 | Valid |
| 3 | 0.396 | 0.361 | Valid |
| 4 | 0.397 | 0.361 | Valid |
| 5 | 0.688 | 0.361 | Valid |
| 6 | 0.572 | 0.361 | Valid |
| 7 | 0.622 | 0.361 | Valid |
| 8 | 0.576 | 0.361 | Valid |
| 9 | 0.631 | 0.361 | Valid |
| 10 | 0.502 | 0.361 | Valid |
| 11 | 0.486 | 0.361 | Valid |
| 12 | 0.637 | 0.361 | Valid |
| 13 | 0.419 | 0.361 | Valid |
| 14 | 0.390 | 0.361 | Valid |
| 15 | 0.700 | 0.361 | Valid |
| 16 | 0.628 | 0.361 | Valid |

**S7. The reliability test of research questionnaires**

| **Variable** | **Reliability Index** | **Critical Value** | **Reliability** |
| --- | --- | --- | --- |
| Health Literacy | 0.711 | 0.7 | Reliable |
| Disease Knowledge | 0.822 | 0.7 | Reliable |
| Self-Care Behavior | 0.818 | 0.7 | Reliable |

**S8. Health literacy questionnaire**

| **Question** | **Answer** | | | | |
| --- | --- | --- | --- | --- | --- |
|  | **Not confident** | **Slightly confident** | **Moderately confident** | **Confident** | **Very confident** |
| How confident are you in filling out forms from the hospital? |  |  |  |  |  |
| **Question** | **Never** | **Rarely** | **Sometimes** | **Often** | **Always** |
| How often do other people help you read hospital documents? (For example, information from a doctor and drug information labels). |  |  |  |  |  |
| How often do you have trouble learning about medical conditions because of difficulty reading hospital documents? (For example, information from a doctor and drug information labels). |  |  |  |  |  |

**S9. Disease knowledge questionnaire**

**For Sections, 1-5, please answer ‘True’, ‘False’ or ‘I don’t know’ to the following questions:**

**Section 1**

| **No.** | **Question** | **True** | **False** | **I don’t know** |
| --- | --- | --- | --- | --- |
| 1. | A person can lead a normal life with one healthy kidney. | □ | □ | □ |
| 2. | Herbal supplements can be effective in treating chronic kidney disease. | □ | □ | □ |
| 3. | Certain medications can help to slow-down the worsening of chronic kidney disease. | □ | □ | □ |

**Section 2** What functions do the kidney perform in our body?

| **No.** | **Question** | **True** | **False** | **I don’t know** |
| --- | --- | --- | --- | --- |
| 4. | The kidneys make urine. | □ | □ | □ |
| 5. | The kidneys clean blood. | □ | □ | □ |
| 6. | The kidneys help to keep blood sugar level normal. | □ | □ | □ |
| 7. | The kidneys help to maintain blood pressure. | □ | □ | □ |
| 8. | The kidneys help to breakdown protein in the body. | □ | □ | □ |
| 9. | The kidneys help to keep the bones healthy. | □ | □ | □ |

**Section 3** Which of the following are commonly used to determine the health of your kidneys?

| **No.** | **Question** | **True** | **False** | **I don’t know** |
| --- | --- | --- | --- | --- |
| 10. | A blood test. | □ | □ | □ |
| 11. | A urine test. | □ | □ | □ |
| 12. | A faecal (poo) test. | □ | □ | □ |
| 13. | Blood pressure monitoring. | □ | □ | □ |

**Section 4** What are the risk factors for chronic kidney disease?

| **No.** | **Question** | **True** | **False** | **I don’t know** |
| --- | --- | --- | --- | --- |
| 14. | Diabetes. | □ | □ | □ |
| 15. | Being female. | □ | □ | □ |
| 16. | High blood pressure. | □ | □ | □ |
| 17. | Heart problems such as heart failure or heart attack. | □ | □ | □ |
| 18. | Excess stress. | □ | □ | □ |
| 19. | Obesity. | □ | □ | □ |

**Section 5** What are the signs and symptoms that a person might have if they have advanced chronic kidney disease or kidney failure?

| **No.** | **Question** | **True** | **False** | **I don’t know** |
| --- | --- | --- | --- | --- |
| 20. | Water retention (excess water in the body). | □ | □ | □ |
| 21. | Fever. | □ | □ | □ |
| 22. | Nausea/vomiting. | □ | □ | □ |
| 23. | Loss of appetite. | □ | □ | □ |
| 24. | Increased fatigue (tiredness). | □ | □ | □ |

**S10. Self-care behavior questionnaire**

| **Statement** | | **Answer** | | | | |
| --- | --- | --- | --- | --- | --- | --- |
|  |  | **Never** | **Rarely** | **Some-times** | **Often** | **Always** |
| **Medication Adherence** | I changed the prescribed dosage times. |  |  |  |  |  |
|  | I stopped taking my medication. |  |  |  |  |  |
|  | I did not take my medication regularly. |  |  |  |  |  |
|  | I changed the frequency of my prescribed medication doses. |  |  |  |  |  |
|  | I changed the prescribed dosage. |  |  |  |  |  |
| **Diet Control** | I adhere to the principles of a kidney disease diet when eating. |  |  |  |  |  |
|  | I consume adequate amounts of food based on the recommendations of a healthcare professional. |  |  |  |  |  |
|  | I control my food and nutrition intake. |  |  |  |  |  |
|  | I adhere to the principles of a kidney disease diet even when on vacation or eating out. |  |  |  |  |  |
| **Exercise** | I exercise regularly. |  |  |  |  |  |
|  | I try to exercise to manage my kidney disease even when I do not feel like it. |  |  |  |  |  |
|  | I make time to exercise even when I'm busy. |  |  |  |  |  |
| **Smoking Habits** | I do not smoke every day. |  |  |  |  |  |
|  | I avoid people or areas where smoking occurs. |  |  |  |  |  |
| **Blood Pressure Monitoring** | I monitor my blood pressure. |  |  |  |  |  |
|  | I can increase the frequency of my blood pressure monitoring, especially when I'm feeling unwell. |  |  |  |  |  |

**S11. Distribution of correct answers to each item of the knowledge questionnaire**

| **No.** | **Question** | **Correct Answer** | | |
| --- | --- | --- | --- | --- |
|  |  | **N** | **(%)** | |
| 1.* | A person can lead a normal life with one healthy kidney. | 80 | | 72.7 |
| 2. | Herbal supplements can be effective in treating chronic kidney disease. | 54 | | 49.1 |
| 3.* | Certain medications can help to slow-down the worsening of chronic kidney disease. | 75 | | 68.2 |
| What functions do the kidney perform in our body? | |  | |  |
| 4.* | The kidneys make urine. | 95 | | 86.4 |
| 5.* | The kidneys clean blood. | 98 | | 89.1 |
| 6. | The kidneys help to keep blood sugar level normal. | 6 | | 5.5 |
| 7.* | The kidneys help to maintain blood pressure. | 94 | | 85.5 |
| 8. | The kidneys help to breakdown protein in the body. | 3 | | 2.7 |
| 9.* | The kidneys help to keep the bones healthy. | 78 | | 70.9 |
| Which of the following are commonly used to determine the health of your kidneys? | |  | |  |
| 10.* | A blood test. | 97 | | 88.2 |
| 11.* | A urine test. | 101 | | 91.8 |
| 12. | A faecal (poo) test. | 25 | | 22.7 |
| 13.* | Blood pressure monitoring. | 85 | | 77.3 |
| What are the risk factors for chronic kidney disease? | |  | |  |
| 14.* | Diabetes. | 106 | | 96.4 |
| 15. | Being female. | 30 | | 27.3 |
| 16.* | High blood pressure. | 107 | | 97.3 |
| 17.* | Heart problems such as heart failure or heart attack. | 86 | | 78.2 |
| 18. | Excess stress. | 7 | | 6.4 |
| 19.* | Obesity. | 73 | | 66.4 |
| What are the signs and symptoms that a person might have if they have advanced chronic kidney disease or kidney failure? | |  | |  |
| 20.* | Water retention (excess water in the body). | 109 | | 99.1 |
| 21. | Fever. | 14 | | 12.7 |
| 22.* | Nausea/vomiting. | 102 | | 92.7 |
| 23.* | Loss of appetite. | 104 | | 94.5 |
| 24.* | Increased fatigue (tiredness). | 107 | | 97.3 |

*Correct item

**S12. Normality test on the domains of adherence, diet, exercise, smoking habits, blood pressure monitoring and total SCB**

| **Variabel** | ***p-value*** |
| --- | --- |
| Total SCB  Domain  Adherence  Diet  Exercise  Smoking Habits  Blood Pressure Monitoring | 0.200  0.200  0.075  0.200  0.200  0.200 |

**S13. Heteroscedasticity test on the domains of adherence, diet, exercise, smoking habits, blood pressure monitoring and total SCB**

| **Independent Variable** | **Total SCB** | **Adherence** | **Diet** | **Exercise** | **Smoking Habits** | **Blood Pressure Monitoring** |
| --- | --- | --- | --- | --- | --- | --- |
|  | ***p-value*** | | | | | |
| HL | 0.020* | 0.479 | - | - | 0.423 | - |
| DK | 0.729 | 0.016* | 0.174 | 0.435 | 0.745 | 0.055 |
| Age | - | 0.002* | 0.443 | 0.723 | - | - |
| Gender | 0.266 | - | 0.774 | - | 0.007* | 0.454 |
| Marital Status | - | 0.210 | - | - | - | 0.196 |
| Education | 0.286 | 0.042* | - | 0.280 | 0.775 | 0.412 |
| Income | 0.846 | 0.669 | - | 0.969 | - | 0.079 |
| Occupation | - | 0.647 | - | 0.283 | 0.473 | - |
| Comorbidities | - | - | - | 0.344 | - | - |
| BMI | - | - | - | 0.310 | <0.001* | 0.115 |
| Blood Pressure | - | - | - | - | - | 0.835 |
| Dialysis Status | 0.865 | - | - | 0.470 | 0.040* | 0.771 |
| Duration of Dialysis | - | 0.788 | - | - | - | - |
| Type of Medication | 0.883 | - | - | - | - | - |
| Routes of Drug Administration | - | - | - | - | - | 0.446 |

*p-value < 0.05

**S14. Multicollinearity test on the domains of adherence, diet, exercise, smoking habits, blood pressure monitoring and total SCB**

| **Independent Variable** | **Total SCB** | | **Adherence** | | **Diet** | | **Exercise** | | **Smoking Habits** | | **Blood Pressure Monitoring** | |
| --- | --- | --- | --- | --- | --- | --- | --- | --- | --- | --- | --- | --- |
|  | **Tolerance** | **VIF** | **Tolerance** | **VIF** | **Tolerance** | **VIF** | **Tolerance** | **VIF** | **Tolerance** | **VIF** | **Tolerance** | **VIF** |
| HL | 0.952 | 1.050 | 0.942 | 1.061 | - | - | - | - | 0.957 | 1.045 | - | - |
| DK | 0.880 | 1.137 | 0.854 | 1.170 | 0.984 | 1.016 | 0.876 | 1.142 | 0.898 | 1.114 | 0.901 | 1.110 |
| Age | - | - | 0.841 | 1.188 | 0.983 | 1.017 | 0.807 | 1.240 | - | - | - | - |
| Gender | 0.927 | 1.079 | - | - | 0.997 | 1.003 | - | - | 0.724 | 1.382 | 0.932 | 1.073 |
| Marital Status | - | - | 0.906 | 1.104 | - | - | - | - | - | - | 0.860 | 1.162 |
| Education | 0.661 | 1.512 | 0.705 | 1.419 | - | - | 0.654 | 1.528 | 0.725 | 1.380 | 0.579 | 1.728 |
| Income | 0.747 | 1.339 | 0.651 | 1.536 | - | - | 0.621 | 1.611 | - | - | 0.712 | 1.405 |
| Occupation | - | - | 0.752 | 1.330 | - | - | 0.714 | 1.400 | 0.694 | 1.440 | - | - |
| Comorbidities | - | - | - | - | - | - | 0.881 | 1.134 | - | - | - | - |
| BMI | - | - | - | - | - | - | 0.951 | 1.051 | 0.985 | 1.015 | 0.891 | 1.122 |
| Blood Pressure | - | - | - | - | - | - | - | - | - | - | 0.796 | 1.256 |
| Dialysis Status | 0.733 | 1.364 | - | - | - | - | 0.697 | 1.435 | 0.748 | 1.336 | 0.642 | 1.558 |
| Duration of Dialysis | - | - | 0.941 | 1.063 | - | - | - | - | - | - | - | - |
| Type of Medication | 0.931 | 1.074 | - | - | - | - | - | - | - | - | - | - |
| Routes of Drug Administration | - | - | - | - | - | - | - | - | - | - | 0.919 | 1.088 |

**S15. Multivariate linear regression analysis of characteristics, DK, HL on total SCB, domains of adherence, diet, exercise, smoking habits and blood pressure monitoring**

| **Variabel** | **Total SCB** | | **Adherence** | | **Diet** | | **Exercise** | | **Smoking Habits** | | **Blood Pressure Monitoring** | |
| --- | --- | --- | --- | --- | --- | --- | --- | --- | --- | --- | --- | --- |
|  | ***β*** | ***p-value*** | ***β*** | ***p-value*** | ***β*** | ***p-value*** | ***β*** | ***p-value*** | ***β*** | ***p-value*** | ***β*** | ***p-value*** |
| HL  Limited (Ref.)  Adequate | 0.679 | 0.713 | 1.318 | 0.018 | - | - | - | - | 0.774 | 0.089 | - | - |
| DK  Low (Ref.)  High | 7.992 | 0.003* | 0.413 | 0.614 | 1.879 | 0.105 | 1.711 | 0.083 | 1.641 | 0.012* | 1.688 | 0.040* |
| Age  18 – 29 years (Ref.)  30 – 49 years  ≥ 50 years | - | - | -0.349  0.507 | 0.642  0.822 | -0.034  0.973 | 0.976  0.402 | -0.548  -1.130 | 0.551  0.272 | - | - | - | - |
| Gender  Male (Ref.)  Female | 4.235 | 0.008* | - | - | 0.844 | 0.221 | - | - | 0.993 | 0.023* | 1.143 | 0.021* |
| Marital Status  Single (Ref.)  Married  Divorced  Widowed | - | - | 0.139  -3.468  0.086 | 0.851  0.003*  0.963 | - | - | - | - | - | - | -0.395  -2.701  -0.245 | 0.632  0.025*  0.902 |
| Education  Elementary School (Ref.)  Junior High School  High School  University | 0.423  3.603  3.653 | 0.878  0.135  0.225 | 0.052  0.704  1.405 | 0.951  0.332  0.115 | - | - | -1.044  -0.454  -0.818 | 0.315  0.619  0.465 | 0.552  0.450  0.909 | 0.421  0.451  0.198 | 0.874  3.257  3.482 | 0.313  <0.001*  <0.001* |
| Income  < RMW (Ref.)  ≥ RMW | 4.946 | 0.027* | 1.118 | 0.126 | - | - | 1.351 | 0.130 | - | - | 1.059 | 0.137 |
| Occupation  Unemployed (Ref.)  Employed | - | - | -1.088 | 0.047* | - | - | 0.624 | 0.364 | -0.950 | 0.039* | - | - |
| Comorbidities  None (Ref.)  Hypertension  Diabetes  Hypertension and diabetes | - | - | - | - | - | - | -0.282  -0.338  -1.977 | 0.734  0.828  0.185 | - | - | - | - |
| BMI  < 18,5 kg/m^2^  18,5 – 24,9 kg/m^2^ (Ref.)  25,0 – 29,9 kg/m^2^  ≥ 30 kg/m^2^ | - | - | - | - | - | - | 0.239  0.482  2.225 | 0.767  0.481  0.152 | 0.560  -0.689  0.965 | 0.298  0.124  0.348 | -0.849  0.052  0.783 | 0.226  0.930  0.555 |
| Blood Pressure  <120/<80 mmHg (Ref.)  120-139/80-89 mmHg  140-159/90-99 mmHg  ≥ 160/ ≥ 100 mmHg | - | - | - | - | - | - | - | - | - | - | 0.052  -1.216  0.378 | 0.954  0.154  0.688 |
| Dialysis Status  Non-dialysis (Ref.)  Hemodialysis  CAPD | 1.881  0.526 | 0.820  0.526 | - | - | - | - | 1.874  1.863 | 0.569  0.583 | -0.683  0.218 | 0.759  0.923 | 2.515  4.262 | 0.362  0.140 |
| Duration of Dialysis  < 6 months (Ref.)  6 – 12 months  > 12 months | - | - | -4.475  -3.129 | 0.039*  0.076 | - | - | - | - | - | - | - | - |
| Type of Medication  Antihypertensive (Ref.)  Antihypertensive, and Other Renal Therapy  Antihypertensive, Antidiabetic, and Other Renal Therapy | -4.789  -0.905  -1.651 | 0.273  0.792  0.808 | - | - | - | - | - | - | - | - | - | - |
| Route of Drug Administration  Oral (Ref.)  Oral and subcutaneous insulin | - | - | - | - | - | - | - | - | - | - | 1.688 | 0.040* |

*p-value < 0.05

RMW: Regional Minimum Wage

CAPD: Continuous Ambulatory Peritoneal Dialysis

**S16. Multivariate linear regression analysis with HC3 (total SCB, domains of adherence, and smoking habits)**

| **Variabel** | **Total SCB** | | | | **Adherence** | | | | **Smoking Habits** | | | |
| --- | --- | --- | --- | --- | --- | --- | --- | --- | --- | --- | --- | --- |
|  | **Multivariate linear regression without HC3** | | **Multivariate linear regression with HC3** | | **Multivariate linear regression without HC3** | | **Multivariate linear regression with HC3** | | **Multivariate linear regression without HC3** | | **Multivariate linear regression with HC3** | |
|  | ***β*** | ***p-value*** | ***β*** | ***p-value*** | ***β*** | ***p-value*** | ***β*** | ***p-value*** | ***β*** | ***p-value*** | ***β*** | ***p-value*** |
| HL  Limited (Ref.)  Adequate | 0.679 | 0.713 | 0.679 | 0.678 | 1.318 | 0.018* | 1.318 | 0.036* | 0.774 | 0.089 | 0.774 | 0,143 |
| DK  Low (Ref.)  High | 7.992 | 0.003* | 7.992 | 0.003* | 0.413 | 0.614 | 0.413 | 0.568 | 1.641 | 0.012* | 1.641 | 0.034* |
| Age  18 – 29 years (Ref.)  30 – 49 years  ≥ 50 years | - | - | - | - | -0.349  0.507 | 0.642  0.822 | -0.349  0.507 | 0.724  0.616 | - | - | - | - |
| Gender  Male (Ref.)  Female | 4.235 | 0.008* | 4.235 | 0.012* | - | - | - | - | 0.993 | 0.023* | 0.993 | 0.030* |
| Marital Status  Single (Ref.)  Married  Divorced  Widowed | - | - | - | - | 0.139  -3.468  0.086 | 0.851  0.003*  0.963 | 0.139  -3.468  0.086 | 0.885  0.025*  0.981 | - | - | - | - |
| Education  Elementary School (Ref.)  Junior High School  High School  University | 0.423  3.603  3.653 | 0.878  0.135  0.225 | 0.423  3.603  3.653 | 0.861  0.168  0.206 | 0.052  0.704  1.405 | 0.951  0.332  0.115 | 0.052  0.704  1.405 | 0.955  0.343  0.127 | 0.552  0.450  0.909 | 0.421  0.451  0.198 | 0.552  0.450  0.909 | 0.461  0.542  0.281 |
| Income  < RMW (Ref.)  ≥ RMW | 4.946 | 0.027* | 4.946 | 0.029* | 1.118 | 0.126 | 1.118 | 0.184 | - | - | - | - |
| Occupation  Unemployed (Ref.)  Employed | - | - | - | - | -1.088 | 0.047* | -1.088 | 0.060 | -0.950 | 0.039* | -0.950 | 0.073 |
| BMI  < 18,5 kg/m^2^  18,5 – 24,9 kg/m^2^ (Ref.)  25,0 – 29,9 kg/m^2^  ≥ 30 kg/m^2^ | - | - | - | - | - | - | - | - | 0.560  -0.689  0.965 | 0.298  0.124  0.348 | 0.560  -0.689  0.965 | 0.205  0.200  0.433 |
| Dialysis Status  Non-dialysis (Ref.)  Hemodialysis  CAPD | 1.881  0.526 | 0.820  0.526 | 1.881  0.526 | 0.734  0.359 | - | - | - | - | -0.683  0.218 | 0.759  0.923 | -0.683  0.218 | 0.886  0.964 |
| Duration of Dialysis  < 6 months (Ref.)  6 – 12 months  > 12 months | - | - | - | - | -4.475  -3.129 | 0.039*  0.076 | -4.475  -3.129 | 0.022*  0.003* | - | - | - | - |
| Type of Medication  Antihypertensive (Ref.)  Antihypertensive, and Other Renal Therapy  Antihypertensive, Antidiabetic, and Other Renal Therapy | -4.789  -0.905  -1.651 | 0.273  0.792  0.808 | -4.789  -0.905  -1.651 | 0.271  0.750  0.696 | - | - | - | - | - | - | - | - |

*p-value < 0.05

RMW: Regional Minimum Wage

CAPD: Continuous Ambulatory Peritoneal Dialysis
